# Mining sorghum pangenome enabled identification of new *dw3* alleles for breeding stable-dwarfing hybrids

**DOI:** 10.1093/g3journal/jkaf054

**Published:** 2025-03-12

**Authors:** Sandeep R Marla, Marcus Olatoye, Matthew Davis, Vincent Otchere, Sarah Sexton-Bowser, Geoffrey P Morris, Terry Felderhoff

**Affiliations:** Department of Agronomy, Kansas State University, Manhattan, KS 66506, USA; Department of Agronomy, Kansas State University, Manhattan, KS 66506, USA; Department of Agronomy, Kansas State University, Manhattan, KS 66506, USA; Department of Agronomy, Kansas State University, Manhattan, KS 66506, USA; Center for Sorghum Improvement, Kansas State University, Manhattan, KS 66506, USA; Department of Soil and Crop Sciences, Colorado State University, Fort Collins, CO 80523, USA; Department of Agronomy, Kansas State University, Manhattan, KS 66506, USA

**Keywords:** sorghum allele mining, stable height, sorghum NAM, trait improvement, haplotype-based markers

## Abstract

Allele mining of crop pangenomes can enable the identification of novel variants for trait improvement, increase crop genetic diversity, and purge deleterious mutations around fixed genomic regions. Sorghum, a C4 cereal crop domesticated in the tropics, was selected for reduced plant height and maturity to develop combine-harvestable and photoperiod-insensitive US grain sorghums. To breed semi-dwarf US grain sorghum hybrids, public and private sector programs mostly used the *dw3-ref* allele, which produces undesirable height revertants (frequency of 0.1–0.3%) due to uneven crossing over at the 882 bp tandem duplication region. Although the *dw3-ref* allele produces revertants, US sorghum breeding programs continued using this allele in the absence of identified allelic variants that suppress revertants. In this study, we leveraged a sorghum pangenome resource (resequenced sorghum association panel and a global diversity panel of 1,661 lines) to identify 7 loss-of-function variants in the *Dw3* gene using the SnpEff variant calling prediction. We validated the Segaolane *dw3* loss-of-function variant, resulting from a 137 bp deletion in the third exon, to suppress revertant production. Segaolane nested association mapping family recombinant inbred line (RILs) with the *dw3-ref* allele produced revertants while no revertants were observed in RILs with the Segaolane *dw3* allele. The availability of resequencing data enabled the designing of haplotype-based markers detecting the Segaolane stable *dw3* allele for marker-assisted trait introgression into elite sorghum breeding lines. This research mining new stable-dwarfing *dw3* alleles demonstrated the application of sorghum pangenome for trait improvement and developing marker-assisted breeding strategies.

## Introduction

Genetic sweeps with alleles for domestication and improved traits reduce genetic diversity in major agronomic crops (Olsen *et al*. 2006; Asano *et al*. 2011; Hufford *et al*. 2012; Lin *et al*. 2014; Moyers *et al*. 2018). For example, the rice *semi-dwarf1* green revolution allele reduced genetic diversity in genomic regions with genes linked to the null semi-dwarfing allele (Asano *et al*. 2011). In tomato, the linkage of allelic variants associated with larger fruit size reduced the accumulation of beneficial glycoalkaloid compounds in the fruit due to hitchhiking of nearby nonfunctional ɑ-tomatine genes with large-size alleles (Zhu *et al*. 2018). Loss of genetic diversity at selected genomic regions necessitates breeding efforts to incorporate diversity at fixed loci. Novel allelic variation at fixed genomic regions, either generated by chemical mutagenesis or CRISPR-Cas gene-editing, or native polymorphisms identified in a pangenome (de novo assemblies or resequenced genomes of a given species), can be used for breeding climate-resilient crops.

Sorghum, a cereal crop domesticated in the tropics, grows up to ∼3 m in height and requires <12 h of daylight for flowering initiation (Quinby and Karper 1945, 1954). Sorghum, introduced into the United States ∼200 years ago, was susceptible to lodging at maturity due to its tall stature and late flowering in temperate climates as a photoperiod-sensitive plant. In the United States, sorghum breeders used nonfunctional dwarfing (*Dw1*–*Dw4*) (Karper and Quinby 1946; Quinby and Karper 1954) and photoperiod-insensitive (*Ma1–Ma6*) (Quinby and Karper 1945; Quinby 1967) alleles to develop semi-dwarf sorghums for combine harvestability, and photoperiod-insensitivity, respectively. To increase the genetic diversity of introduced US sorghums, the US sorghum conversion (SC) and reinstated SC programs converted ∼700 exotic progenitor lines to semi-dwarf and photoperiod-insensitive sorghums by crossing with a 4-dwarf photoperiod-insensitive temperate sorghum line BTx406 (Stephens *et al*. 1967; Klein *et al*. 2016; Horne *et al*. 2020). Despite increasing genetic diversity, selection for semi-dwarfing and maturity alleles reduced genetic diversity around these major fixed loci (Thurber *et al*. 2013). To improve reduced genetic diversity around these fixed loci, it is important to identify new alleles in exotic sorghums and introgress into elite sorghums using marker-assisted breeding.

**Fig. 1.**
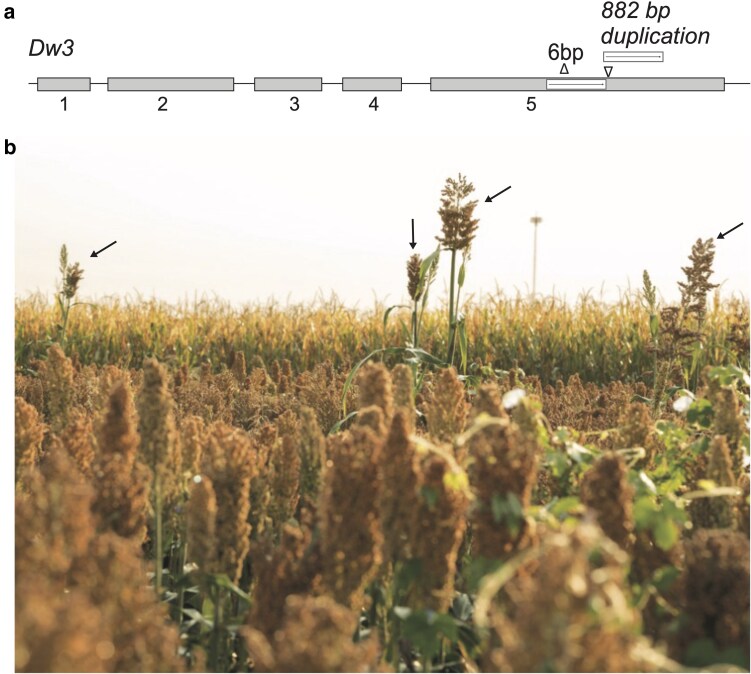
Sorghum plant height *dw3-ref* allele producing undesirable height revertants. a) Gene model of the wildtype *Dw3* gene containing 5 exons. An 882 bp tandem duplication resulted in semi-dwarf plants, this *dw3-ref* allele produces revertants at 1–3% depending on the genetic background. A 6 bp deletion in the fifth exon resulted in the new *dw3-sd2* stable-dwarfing allele. b) Undesirable revertants, indicated by arrows, in a US grain sorghum hybrid field.

SC program was conducted using conventional breeding. Knowledge of the genetic basis of major plant height loci fixed in US sorghums can enable marker-assisted breeding to accelerate precise trait introgression. Genes underlying 3 of the major 4 plant height loci were characterized (Multani *et al*. 2003; Hilley *et al*. 2016, 2017). The *Dw3* plant height gene, cloned based on the maize ortholog *brachytic2* (*br2*) gene, codes for an ABC transporter (Multani *et al*. 2003). In sorghum, the *dw3-ref* nonfunctional allele has been the most commonly used dwarfing allele for breeding programs in the past 60 years. The *dw3-ref* allele resulted from a tandem duplication of the 882-bp sequence in the fifth exon. Despite generating semi-dwarf US grain sorghum hybrids, the *dw3-ref* allele produces undesirable height revertants (with wildtype *Dw3* allele) at a frequency of 0.1–0.3% due to uneven crossing over at the 882-bp tandem duplication (Fig. 1). In sorghum hybrid seed production, manual rouging of the revertants increases seed costs, which otherwise can pollinate female lines with the *dw3-ref* allele. After utilizing the *dw3-ref* allele in sorghum breeding for 60 years, a new *dw3* allele (*dw3-sd2* in the sorghum line Tx2737) lacking the 882-bp duplication but containing a 6-bp deletion in the fifth exon was identified as a genetic resource for developing stable dwarfing sorghums (Barrero Farfan *et al*. 2012).

To develop stable dwarfing sorghums and increase the genetic diversity around the *dw3* locus fixed in the US grain sorghums, we leveraged resequencing data of the sorghum association panel (SAP) and a global diversity panel of 1,661 lines to identify new dwarfing alleles using the gene-first/reverse genetics approach. As the occurrence of *dw3-ref* allele revertants is caused by unequal crossover at the 882 bp tandem duplication, we hypothesized complete loss-of-function or null alleles in the *Dw3* gene inhibit revertant production in the US grain sorghum hybrids. The main objectives of this research were (1) mine sorghum pangenome to identify new complete loss-of-function *dw3* alleles, (2) validate if a complete loss-of-function *dw3* allele inhibits revertant production, and (3) generate molecular markers to introgress a new stable dwarfing allele into US elite grain sorghum lines. In this research, mining sorghum genomes enabled the detection of new *dw3* alleles to develop stable-dwarfing sorghum hybrids and improve genetic diversity at the fixed *Dw3* plant height locus.

## Materials and methods

### Plant materials

Segaolane family recombinant inbred lines (RILs) from the sorghum nested association mapping (NAM) population (Bouchet *et al*. 2017), generated by crossing the sorghum NAM common parent RTx430 (PI655996) with the alternate parent Segaolane (PI656023), were used to determine if the Segaolane dwarfing allele produced revertants. RTx430 is a 3-dwarf sorghum inbred, containing *dw1Dw2dw3dw4* alleles, and Segaolane is a 2-dwarf sorghum based on plant height and KASP genotyping. A subset of the Segaolane family (*n* = 87, <160 cm height), 29 RILs containing the RTx430 *dw3*-*ref* haplotype, and 58 RILs with the Segaolane haplotype were evaluated for revertants (Supplementary Table 1). A Segaolane RIL (PI697849/PR1314_3429), containing the *dw3-sd6* allele and desirable agronomics, was used to conduct genetic crosses with elite lines for marker-assisted introgression into the US elite sorghum breeding lines. In addition to *dw3-sd6*, stable-*dw3* alleles from sorghum lines SC155, SC1424, and SC473 were evaluated for revertant production in the 2024 field season.

### Mining for *dw3* complete loss-of-function alleles

Mining *dw3* null alleles was conducted using the whole-genome resequencing data of the SAP (Boatwright *et al*. 2022) and a global diversity panel of 1,661 lines (unpublished data from our collaborator Dr. Geoffrey Morris's lab). Resequenced genomes of diverse sorghum lines were aligned to the sorghum reference genome BTx623 v3.0 (McCormick *et al*. 2018). The SnpEff program (Cingolani *et al*. 2012) was used to identify complete loss-of-function alleles in the *Dw3* gene. Alleles with a high SnpEff effect, resulting from null variants, were included in this manuscript (Supplementary Table 2). To analyze the global distribution of the *dw3* null alleles, we built 2 principal component analysis (PCA) axes with previously published *Ape*KI GBS data of 401 global sorghum accessions (Morris *et al*. 2013; Hu *et al*. 2019) using the prcomp function in R. PCA results were plotted using the R plot.

### Field phenotyping for revertants

A selected subset of Segaolane family RILs (*n* = 87), selected based on the haplotype and short stature (>160 cm) from the Manhattan 2015 field season, were planted in 2022 in Manhattan, Kansas. The controls were sorghum lines BTx623, RTx430, Segaolane, and the commercial hybrid DKS36-07. In this field study, each RIL was planted in 2-row plots with each row containing 50 seeds, planted in 10-feet rows with 3-feet alleys between ranges and a spacing of 2.5 feet between the rows. Each RIL was planted in 3 replications and the RILs were completely randomized within the block. Phenotyping for revertants was conducted after flowering. Leaf tissue samples were collected from the revertants and a semi-dwarf representative plant in each RIL for genotyping. In 2024, strip plots of lines SC155, SC1424, and SC473 were planted to evaluate whether the predicted *dw3* null alleles produced revertants.

### *Dw3* amplification and Sanger sequencing

Overlapping PCR primers were designed to amplify the *Dw3* allele from Segaolane. For stabilizing the PCR reaction at a higher annealing temperature (62°C), 50% glycerol and 25 mM MgCl_2_ were added to the master mix. Amplified PCR products were separated on a 2% agarose gel to visualize the third exon size difference between BTx623 and Segaolane. Eight overlapping primer pairs were used to amplify the Segaolane *Dw3* allele (Supplementary Table 3). A diagnostic primer pair was designed to amplify the fifth exon 882 bp duplication. Molecular markers generated to amplify *Dw3* were ordered from Eurofins Genomics. The PCR product purification and Sanger sequencing were conducted at GENEWIZ (South Plainfield, NJ).

### Composite interval mapping

Sorghum NAM plant height from the Manhattan 2015 field trial was used for joint linkage mapping (JLM) analysis (Bouchet *et al*. 2017). Composite interval mapping (CIM) of plant height in individual families was not included in the sorghum NAM publication. To map plant height QTL in individual NAM families, CIM was conducted with individual sorghum NAM families with previously published plant height data from Manhattan in 2015. A linkage map for the NAM population was generated using the R/qtl package (Broman 2015) (Supplementary Table 4). CIM with R/qtl was used for performing linkage mapping and significant QTL were determined based on the threshold level defined by computing 1,000 permutations. CIM was performed using plant height data from the MN2015 field season.

### Marker development and validation

Two KASP markers were designed at Intertek, a technology provider for the Excellence in Breeding (EiB) project, to detect the *dw1* and *dw2* dwarfing alleles. To develop KASP markers at Intertek, 200 bp flanking sequence of the genotyping variant was submitted (File S1). In the *Dw3* gene, the presence or absence of the 882-bp tandem duplication in the fifth exon (*dw3-ref* allele) was determined by PCR using a marker between the tandem duplications (Supplementary Table 2). Considering the large insertion in the *dw3-ref* allele, SNPs and indels flanking 200 Kb from the *Dw3* gene were extracted using the Samtools HTSlib program (Bonfield *et al*. 2021; Danecek *et al*. 2021) to aid in the design of KASP markers flanking the *dw3-ref* allele. SNP/indel data visualization and linkage disequilibrium (LD) analysis between SNPs was conducted using the TASSEL package (Bradbury *et al*. 2007). Indels and SNPs detected from the resequencing data of the SAP and 1,661 panels were utilized to generate a *dw3-ref* KASP marker (snpSB00646).

## Results

### Mining sorghum resequencing data identified 7 *dw3* complete loss-of-function alleles

**Fig. 2.**
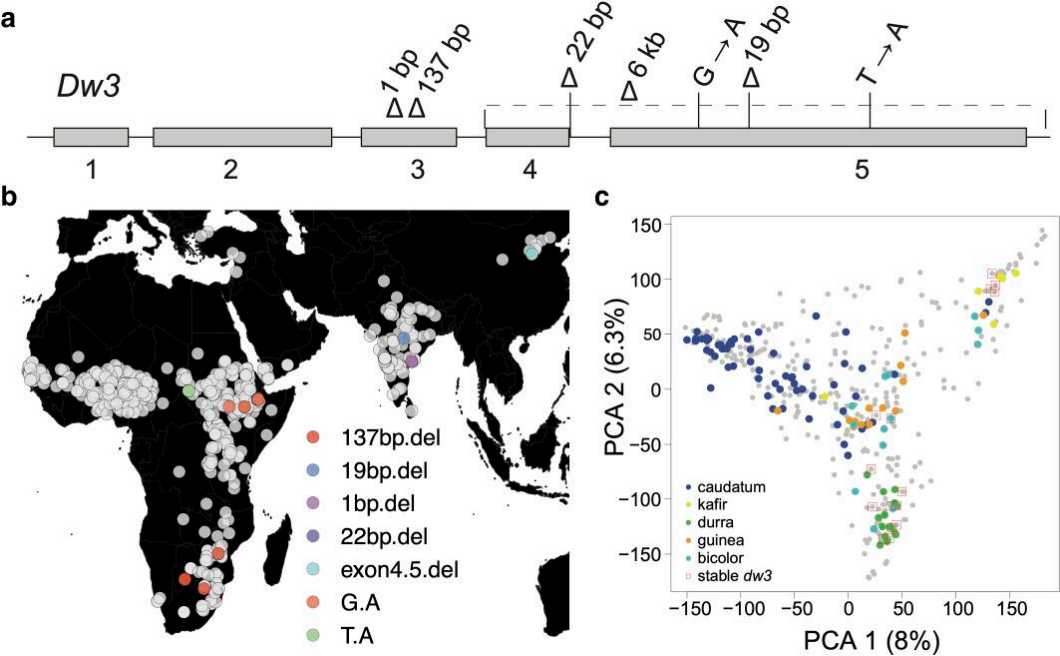
Global distribution of *dw3* null alleles. a) New dwarfing alleles at the *dw3* locus identified using SnpEff assay of the sorghum pangenome. Deletions and SNPs are indicated in the *Dw3* gene model. b) Global distribution of *dw3* null alleles. c) PCA of the sorghum association global diversity panel. Major botanical races (caudatum, kafir, durra, Guinea, and bicolor) of global accessions are indicated with different colors.

To identify *dw3* null variants using reverse genetics/gene-first approach, SnpEff variant calling prediction was conducted using the resequencing data of the SAP and a global diversity panel of 1,661 lines. Allele mining identified 7 *dw3* complete loss-of-function alleles (Fig. 2a and Supplementary Table 2). We identified a deletion of 137 bp in the third exon, predicted to produce a truncated protein, in Segaolane (PI656023), SC757/Marupantse (PI576352), SC673 (PI576339), and SC648 (PI533955) (File S1 and S2). A large deletion of ∼6 kb spanning the fourth and fifth exons, and the 3′ UTR region was observed in the Chinese lines Shan Qui Red (PI656025) and San Chi San (PI542718). SC937 (PI576348) contained a deletion of 19 bp in the fifth exon that generated a premature stop codon. A single nucleotide substitution (G/A) in SC155/R3-80 (PI534155), IS11270 (PI329518), IS11271 (PI329519), SC6/OrangeNo.l_Baijo (PI533902), and IS12646 (PI276816) in the fifth exon was predicted to produce a truncated protein. Deletion of a single nucleotide in the third exon, generating a truncated protein, was identified in lines SC199/Karad 40581 (PI533810), SC473/Jonar Tamargundi (PI534028), SC480 (PI656097), SC498 (PI656099), and SC500 (PI656100). In SC1424 (PI656078), a 22 bp deletion at the intron-exon junction of the fourth exon and fourth intron resulted in a truncated protein. In IS19026 (PI569050), a SNP (T/A) in the fifth exon was predicted to produce a truncated protein.

### Global distribution indicates multiple origins of *dw3* null alleles

To breed semi-dwarf varieties or hybrids, sorghum breeders select parental lines with reduced height without genetic information of the existing dwarfing alleles. Geographical distribution of new dwarfing alleles showed *dw3* complete loss-of-function alleles in sorghum lines from China (2 lines), Ethiopia (6 lines), South Africa (1 line), Botswana (2 lines), Zimbabwe (1 line), India (5 lines), Sudan (1 line), and Mali (1 line) (Fig. 2b and Supplementary Table 2). Similar *dw3* null alleles were present within a geographical location, and the presence of the same allele in different landraces indicates a single origin event for a given location (Fig. 2b). In contrast, *dw3* null alleles differed between geographical locations. In the major sorghum races, we observed stable dwarfing alleles in durra, kafir, bicolor, and guinea races, while no dwarfing alleles were identified in the caudatum race sorghums (Fig. 2c).

### Deletion of 137 bp in the third exon resulted in the dwarfing *dw3-sd6* allele

**Fig. 3.**
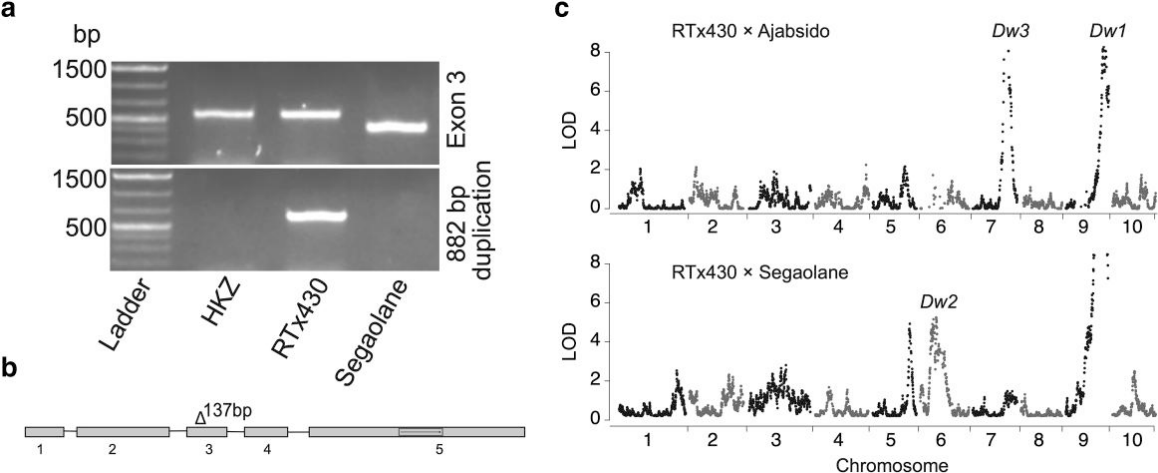
Genetic characterization of the Segaolane *dw3-sd6* allele. a) Gel electrophoresis image (top panel) depicting the presence of 137 bp deletion in the third exon in Segaolane but no deletion in the tall Chinese line Hong Ke Zi (HKZ) with the wildtype *Dw3* and an elite sorghum line RT430 containing the *dw3-ref* allele. PCR amplification in the bottom panel indicates the presence of the *dw3-ref* allele in RTx430 and the lack of tandem duplication in HKZ and Segaolane. b) The *Dw3* gene model depicting 137 bp deletion in the Segaolane *dw3-sd6* allele. c) CIM of sorghum plant height in Ajabsido and Segaolane NAM families. Mapping of the *Dw3* QTL in the RTx430 × Ajabsido family indicates the presence of functional *Dw3* in Ajabsido. The failure to map *Dw3* in the RTx430 × Segaolane family indicates the Segaolane line contains a nonfunctional *dw3* allele.

Given the availability of the sorghum NAM Segaolane family (Bouchet *et al*. 2017), we conducted detailed genetic analyses of the Segaolane 137 bp loss-of-function variant to determine whether this null allele inhibited revertant production. Confirming the 137 bp deletion in Segaolane, PCR amplification of the third exon from Segaolane revealed a ∼100 nt smaller amplicon than the *Dw3* allele from HKZ, a tall Chinese line, and RTx430 containing the *dw3*-*ref* allele (Fig. 3a). Sanger sequencing of the third exon validated the 137 bp deletion in Segaolane, compared to the WT *Dw3* and *dw3-ref* alleles (Fig. 3b). Additionally, molecular markers designed to amplify the *dw3-ref* 882 bp duplication failed to amplify in Segaolane and HKZ while producing a 600 bp amplicon with RTx430, indicating the absence of the 882 bp duplication in the Segaolane *dw3* allele (Fig. 3a). Sanger sequencing of the Segaolane *dw3* allele, to identify other DNA changes in the *dw3* allele impacting the gene function, revealed 8 SNPs in the fifth exon that resulted in missense mutations to the predicted protein. In summary, 137 bp deletion in the third exon and the absence of 882 bp duplication in the *dw3-ref* allele indicated the Segaolane dwarfing allele as a new dwarfing allele. Given that previously published Barrero Farfan *et al*. (2012) and a recently published research by Diatta-Holgate *et al*. (2024) named stable dwarfing alleles *dw3-sd2 to dw3-sd5*, respectively, we designated the Segaolane *dw3* dwarfing allele as *dw3-sd6*.

### Linkage mapping failed to detect the *Dw3* plant height QTL in the Segaolane family

Given that RTx430 contains a nonfunctional *dw3-ref* allele (*dw1Dw2dw3*), we predicted CIM with the RTx430 × Segaolane family will not detect the *dw3* QTL if Segaolane contains a nonfunctional allele. JLM analysis was conducted for plant height with the sorghum NAM population (Bouchet *et al*. 2017); however, CIM for individual bi-parental families was not reported previously. CIM with the plant height data from Manhattan 2015 mapped QTL at *Dw1*, *Dw2*, or *Dw3* loci in different NAM families (Supplementary Fig. 1). The Ajabsido family mapped *Dw3* and *Dw1* plant height QTL, while the Segaolane family mapped *Dw2* and *Dw1* QTL (Fig. 3c). Supporting our prediction, no plant height QTL was identified with the Segaolane family at the *dw3* locus, suggesting the 137 bp deletion in the Segaolane *Dw3* gene resulted in a nonfunctional *dw3* allele.

### Revertants in the Segaolane family contained the *dw3-ref* allele

**Fig. 4.**
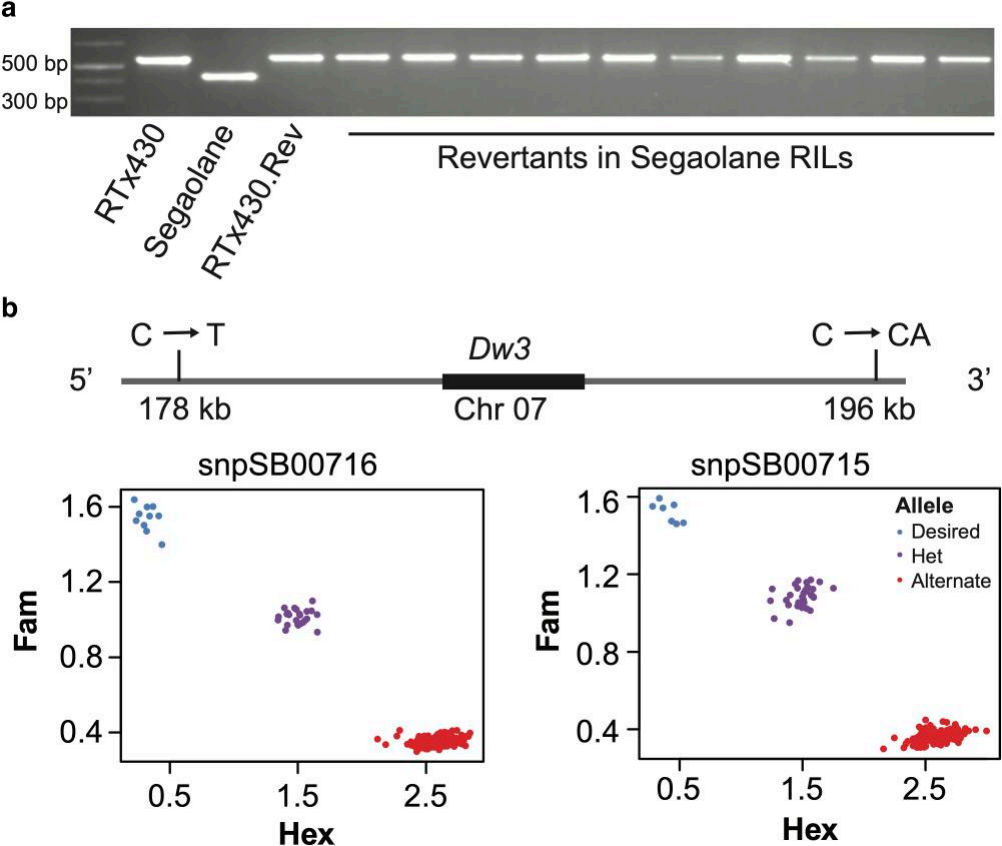
Marker-assisted introgression of the stable-dwarfing *dw3-sd6* allele. a) PCR evaluation of the third exon from RTx430, Segaolane, a revertant from the RTx430 inbred (RTx430.Rev), and 10 revertants in RILs from the Segaolane family. Segaolane *dw3-sd6* allele with the 137 bp deletion in the third exon produced a smaller amplicon compared to RTx430, RTx430.Rev, and the 10 revertants indicating all the revertants were generated by the *dw3-ref* allele and Segaolane *dw3-sd6* to be a stable-dwarfing allele. b) Resequencing of the SAP and global diversity panel of 1,661 lines enabled the identification of flanking SNPs for KASP marker development. Two KASP markers (snpSB00715 and snpSB00716) were designed to introgress the Segaolane stable-dwarfing *dw3-sd6* allele into elite US grain sorghum lines. Desired and alternate alleles were differentiated through the competitive binding of 2 allele-specific forward primers. The forward primers contained a unique tail sequence that corresponded with 2 universal FRET (fluorescence resonant energy transfer) cassettes, 1 labeled with FAM dye and the other with HEX dye. Homozygous desired allele was colored in blue, homozygous alternate allele in red, and the heterozygotes were indicated in purple.

In the Manhattan 2022 (MN2022) field trial with the Segaolane family RILs (*n* = 85), we obtained 5,708 plants with the *dw3-ref* allele and 11,484 plants with the *dw3-sd6* allele. We observed 10 revertants in the MN2022 field trial. Revertants and semi-dwarf plants in the RIL family contained similar grain color, plant color, and midrib color but differed in plant height, leaf angle, and panicle architecture. Given the *dw3-sd6* allele with a 137 bp deletion in the third exon is considered a stable dwarfing allele; we predicted the Segaolane family RILs with RTx430 *dw3-ref* allele to generate all the revertants. PCR assay of the third exon showed a larger fragment in RTx430 and a revertant plant in the RTx430 inbred compared to Segaolane with the 137 bp deletion, indicating RTx430 and the RTx430 revertant to contain the *dw3-ref* allele (Fig. 4a and Supplementary Table 5). All 10 revertants in the Segaolane RILs contained a larger amplicon, similar to RTx430, indicating revertant production by the *dw3-ref* allele. The frequency of revertants identified (10/5708 = 0.18%) in this study was similar to the *dw3-ref* allele revertant frequencies reported in previous studies (Barrero Farfan *et al*. 2012; Diatta-Holgate *et al*. 2024).

### KASP marker-enabled breeding of elite semi-dwarf sorghums

Two KASP markers developed to detect the *dw1* (A→T, Sbv3.1_09_57040002W, snpSB00493) and *dw*2 (GA/–, Sbv3.1_06_42806045I, snpSB00494) causal variants successfully differentiated the desired dwarfing allele from the alternate allele (Supplementary Fig. 2). To detect the *dw3-ref* allele to replace it with a stable-dwarfing allele, a SNP (G/A) within the *Dw3* gene used to detect the *dw3-ref* allele (Sbv3.1_07_59821995R snpSB00646) determined the presence of the *dw3-ref* allele (Supplementary Fig. 2).

Given the *dw3-sd6* allele resulted from a 137 bp deletion and the other SNPs in the *dw3-sd6* allele were shared among other sorghum lines with functional *Dw3*/*dw3-ref* alleles, it was not possible to generate high-throughput KASP markers tracking the *dw3-sd6* causative allele. Therefore, we designed KASP markers using 1 indel [C/CA; 196 kb 3′ of *Dw3*] and a SNP [C/T; 178 kb 5′ of *Dw3*] in linkage and LD with the *dw3-sd6* allele to differentiate the desired Segaolane *dw3-sd6* allele vs the alternate *Dw3*/*dw3-ref* allele. We developed 2 KASP markers based on the flanking polymorphisms Sbv3.1_07_60018674I (snpSB00715) and Sbv3.1_07_59651093Y (snpSB00716) (Fig. 4b). The KASP markers on a segregating population showed 3 clusters: homozygous for the *dw3-sd6* allele, homozygous for the alternate allele, and a heterozygous cluster (Fig. 4b). Testing snpSB00715 and snpSB00716 with 15 diverse sorghum lines showed its ability to identify the targeted allele in different sorghum lines. The allelic frequency of the SNPs used in marker development showed them to be rare alleles (1%) in the pangenome data, indicating the functionality of this marker in diverse breeding lines from different breeding programs. Flanking markers on both sides of the *dw3-sd6* allele assisted in introgressing the desired allele into elite breeding lines for developing stable-dwarfing sorghum hybrids.

## Discussion

### Pangenome-enabled identification of new stable dwarfing alleles

In this study, we provide an example of leveraging publicly available genomic and genetic resources for identifying new alleles, rapid hypothesis testing, and developing high-throughput genotyping markers for trait improvement. Reference quality de novo sequencing of 32 sorghum lines (sorghum pangenome, available on Phytozome) and resequencing data of the 1,661 panel, US grain SAP of 400 lines (Boatwright *et al*. 2022), and a worldwide collection of 499 lines (Lozano *et al*. 2021) are useful genomics resources to identify novel alleles and design molecular breeding strategies for trait improvement. Although previous GBS data provided improved mapping resolution (Morris *et al*. 2013; Bouchet *et al*. 2017; Hu *et al*. 2019), low marker density impeded mining novel allelic variants in the gene of interest.

The most commonly used dwarfing allele (*dw3-ref*) at the *dw3* locus produces revertants in US grain sorghum hybrids (Multani *et al*. 2003). Based on the genetics of sorghum revertant production, we hypothesized complete loss-of-function alleles at the *dw3* locus inhibit revertants. Identification of 7 complete loss-of-function variants putatively impacting the *dw3* gene activity was possible by allele mining the resequencing data of the SAP and the 1,661 global diversity panel (Fig. 2a). Given that one of the complete loss-of-function variants was in Segaolane, an alternate parent of the sorghum NAM population, detailed genetic characterization of the Segaolane family RILs rapidly validated the *dw3* null alleles ability to suppress revertants hypothesis (Fig. 3). In addition to the *dw3-sd6* allele, field trials with 3 additional null *dw3* alleles also validated that null *dw3* alleles suppress revertant production (Supplementary Table 6). Supporting our hypothesis is a parallel study demonstrating 3 *dw3* null alleles in the fifth exon inhibiting revertant production in SC124, SC134, and SC991 (Diatta-Holgate *et al*. 2024). Null *dw3* allelic variants in the fifth exon were identified by Sanger sequencing of lines without the tandem duplication and validated their ability to suppress revertants production in an RTx430 BC_2_F_5_ population (Diatta-Holgate *et al*. 2024). Although our research and Diatta-Holgate *et al*. (2024) addressed the same hypothesis, the approaches used to identify *dw3* null alleles, the *dw3* null alleles identified, and the breeding methods for validating the revertant production were different and complementary between these 2 studies.

KASP markers differentiating the desired (causal variant) vs the alternate allele can be designed using SNPs or a few bp indels. Although developing KASP markers for large deletions is tedious, the KASP markers can differentiate the desired vs the alternate alleles (Yerka *et al*. 2024). In the case of indels up to 200 bp, both alleles get amplified by the KASP primers, leading to misinterpretation of the genotyping results. Instead of developing markers targeting the causative alleles, designing haplotype-based markers is the preferred solution for making breeding decisions. Given the availability of resequencing data, we were able to develop haplotype-based KASP markers flanking the Segaolane *dw3-sd6* allele to introgress this stable-dwarfing allele into elite sorghum breeding lines, an endeavor that is currently underway. Low allele frequency (1%) of the SNP and the indel demonstrate them as rare polymorphisms private to the lines with the *dw3-sd6* allele. Overall, our results demonstrate pangenome enabled rapid identification of multiple *dw3* alleles and the development of flanking KASP markers for precise introgression of the stable *dw3-sd6* allele, demonstrating the utility of pangenome for rapid trait improvement.

### Increasing genetic diversity at fixed major loci using novel allelic variants and new dwarfing mechanisms

Reduced sorghum genetic diversity around fixed loci controlling plant height and flowering can impede the climate-resiliency of US grain sorghums (Thurber *et al*. 2013). As most of chromosome 6 was fixed in SC lines, marker-assisted breeding by the reinstated SC program was conducted to increase the genetic diversity of the US sorghums (Klein *et al*. 2016; Horne *et al*. 2020). Given the existence of only 1 dwarfing allele at the *dw1* and *dw2* loci (Grant *et al*. 2023), probable deleterious mutations linked with the only desired allele at the major plant height loci remain a concern. Identifying new dwarfing alleles in diverse genetic backgrounds could increase genetic diversity and purge deleterious mutations at major fixed loci in US grain sorghum. In contrast to the *dw1* and *dw2* loci, 19 loss-of-function variants were identified at the *dw3* locus from our research, Grant *et al*. (2023), and Diatta-Holgate *et al*. (2024). In addition to our study, Diatta-Holgate *et al*. (2024) demonstrated null variants to suppress revertant production. The geographic distribution of the new stable-dwarfing alleles from different countries and sorghum races indicates these alleles can be used in local breeding programs to develop stable-dwarfing sorghum hybrids/varieties. To purge deleterious mutations around the fixed loci, sorghum breeders can utilize different null alleles in the hybrid seed parents.

Considering only 1 *dw1* and *dw2* alleles are used in US sorghums, creating new dwarfing alleles at each locus is critical to increasing genetic diversity. Novel *dw1* and *dw2* dwarfing null alleles can be generated by either ethyl methanesulfonate mutagenesis or precise gene-editing using CRISPR-Cas biotechnology tools. In sorghum, auxin and brassinosteroid pathways were utilized in breeding semi-dwarf sorghum (Multani *et al*. 2003; Hirano *et al*. 2017). In contrast, GA biosynthesis mutants were used for developing dwarf green revolution varieties in rice and wheat (Spielmeyer *et al*. 2002; Pearce *et al*. 2011). GA biosynthesis mutants were not utilized in semi-dwarf sorghum breeding as the null alleles caused sorghum culm bending (Ordonio *et al*. 2014). Like sorghum, maize GA biosynthesis mutants exhibit pleiotropic effects (anther ear production and severe stunting) (Bensen *et al*. 1995). To bypass the negative pleiotropy of the maize GA pathway, hybrid seed companies are modulating the GA biosynthesis gene expression using miRNA-based constructs or identifying weak mutations in the GA signaling pathway (Paciorek *et al*. 2022). Generating weak alleles in GA-signaling genes could provide genetic variation for utilizing the new class of dwarfing genes for sorghum improvement. Overall, combining pangenome mining for allelic variation and modern biotechnology for generating novel alleles can improve genetic variation at fixed loci, develop molecular breeding strategies for precise introgression of desired alleles, and improve climate-resiliency of sorghum hybrids.

## Supplementary Material

jkaf054_Supplementary_Data

## Data Availability

GBS data of the sorghum NAM population and plant height data from MN2015 were previously published in https://doi.org/10.1534/genetics.116.198499 (Bouchet *et al*. 2017). Supplemental Figures: Supplementary Fig. 1 contains composite interval mapping results of MN2015 plant height for NAM individual families. Supplementary Fig. 2 depicts KASP marker genotyping for *dw1*, *dw2*, and *dw3-ref* (haplotype-based markers) alleles. Supplemental Tables contain Supplementary Tables 1–6. Supplementary Table 1 includes selected Segaolane family RILs used for evaluating revertant production. Supplementary Table 2 contains the null *dw3* alleles identified using the SnpEff assay. Supplementary Table 3 contains primers used for *Dw3* amplification and *dw3-ref* allele diagnostic markers. Supplementary Table 4 contains the linkage map generated for the NAM population. Supplementary Table 5 contains revertants generated in the Segaolane family RILs and the *dw3* allele in the revertants determined using PCR markers. Supplementary Table 6 contains the number of plants for each new *dw3* allele evaluated for revertant production. Supplementary File 1 contains the predicted amino acid sequence and 200 bp flanking sequences used for KASP marker development. File S2 includes the SnapGene sequence file of the *dw3-ref* sequence from the reference genome BTx623 and new alleles indicated in the sequence file. Supplemental material available at G3 online.
