# Supplementary material for: Mining sorghum pangenome enabled identification of new dw3 alleles for breeding stable-dwarfing hybrids
Source: G3 (Bethesda). 2025 Mar 12;15(5):jkaf054. doi: 10.1093/g3journal/jkaf054 (PMC12060247; doi:10.1093/g3journal/jkaf054)
Supplement: jkaf054_Supplementary_Data [file jkaf054_supplementary_data.zip › File_S1_G3-2024-405623.pdf]

Predicted amino acid sequence for new *dw3* alleles

Dw3 Sobic.007G163800 Reverse

Allele: *Dw3* wild-type (predicted peptide of 1248 aa)
Lines: HKZ and NSZ
MLVGTLGALVHGCSLPVFLRFFADLVDSFGSHANDPDTMVRLVVKYAFYFLVVGAAIWASSWAEISCWMWTGERQSTRMRIRYLD
AALRQDVSFFDTDVRASDVIYAINADAVVVQDAISEKLGNLIHYMATFVAGFVVGFTAAWQLALVTLAVVPLIAVIGGLSAAALA
KLSSRSQDALSGASGIAEQALAQIRIVQAFVGEEREMRAYSAALAVAQKIGYRSGFAKGLGLGGTYFTVFCCYGLLLWYGGHLVR
GNHTNGGLAIATMFSVMIGGLALGQSAPSMAAFAKARVAAAKIFRIIDHRPGISSRDGEDGGGVELESVTGRVEMRGVDFAYPSR
PDVPILRGFSLSVPAGKTIALVGSSGSGKSTVVSLLERFYDPSAGQILLDGHDLKSLKLRWLRQQIGLVSQEPTLFATSIKENLL
LGRDSQSATQAEMEEAARVANAHSFIVKLPDGYDTQVGERGLQLSGGQKQRIAIARAMLKNPAILLLDEATSALDSESEKLVQEA
LDRFMIGRTTLVIAHRLSTIRKADVVAVLQGGAVSEMGTHDELMAKGENGTYAKLIRMQEQAHEAALVNARRSSARPSSARNSVS
SPIMTRNSSYGRSPYSRRLSDFSTSDFTLSIHDPHHHHRTMADKQLAFRAGASSFLRLARMNSPEWAYALVGSLGSMVCGSFSAI
FAYILSAVLSVYYAPDPRYMKREIAKYCYLLIGMSSAALLFNTVQHVFWDTVGENLTKRVREKMFAAVLRNEIAWFDADENASAR
VAARLALDAQNVRSAIGDRISVIVQNSALMLVACTAGFVLQWRLALVLLAVFPLVVGATVLQKMFMKGFSGDLEAAHARATQIAG
EAVANLRTVAAFNAERKITGLFEANLRGPLRRCFWKGQIAGSGYGVAQFLLYASYALGLWYAAWLVKHGVSDFSRTIRVFMVLMV
SANGAAETLTLAPDFVKGGRAMRSVFETIDRKTEVEPDDVDAAPVPERPKGEVELKHVDFSYPSRPDIQVFRDLSLRARAGKTLA
LVGPSGCGKSSVLALVQRFYEPTSGRVLLDGKDVRKYNLRALRRVVAVVPQEPFLFAASIHDNIAYGREGATEAEVVEAATQANA
HRFISALPEGYGTQVGERGVQLSGGQRQRIAIARALVKQAAIMLLDEATSALDAESERCVQEALERAGNGRTTIVVAHRLATVRN
AHTIAVIDDGKVVEQGSHSHLLKHHPDGCYARMLQLQRLTGGAAPGPPPSSSNGAAA-

Allele: *dw3-ref* (predicted peptide of 1542 aa)
Lines: BTx623 and RTx430
MLVGTLGALVHGCSLPVFLRFFADLVDSFGSHANDPDTMVRLVVKYAFYFLVVGAAIWASSWAEISCWMWTGERQSTRMRIRYLD
AALRQDVSFFDTDVRASDVIYAINADAVVVQDAISEKLGNLIHYMATFVAGFVVGFTAAWQLALVTLAVVPLIAVIGGLSAAALA
KLSSRSQDALSGASGIAEQALAQIRIVQAFVGEEREMRAYSAALAVAQKIGYRSGFAKGLGLGGTYFTVFCCYGLLLWYGGHLVR
GNHTNGGLAIATMFSVMIGGLALGQSAPSMAAFAKARVAAAKIFRIIDHRPGISSRDGEDGGGVELESVTGRVEMRGVDFAYPSR
PDVPILRGFSLSVPAGKTIALVGSSGSGKSTVVSLLERFYDPSAGQILLDGHDLKSLKLRWLRQQIGLVSQEPTLFATSIKENLL
LGRDSQSATQAEMEEAARVANAHSFIVKLPDGYDTQVGERGLQLSGGQKQRIAIARAMLKNPAILLLDEATSALDSESEKLVQEA
LDRFMIGRTTLVIAHRLSTIRKADVVAVLQGGAVSEMGTHDELMAKGENGTYAKLIRMQEQAHEAALVNARRSSARPSSARNSVS
SPIMTRNSSYGRSPYSRRLSDFSTSDFTLSIHDPHHHHRTMADKQLAFRAGASSFLRLARMNSPEWAYALVGSLGSMVCGSFSAI
FAYILSAVLSVYYAPDPRYMKREIAKYCYLLIGMSSAALLFNTVQHVFWDTVGENLTKRVREKMFAAVLRNEIAWFDADENASAR
VAARLALDAQNVRSAIGDRISVIVQNSALMLVACTAGFVLQWRLALVLLAVFPLVVGATVLQKMFMKGFSGDLEAAHARATQIAG
EAVANLRTVAAFNAERKITGLFEANLRGPLRRCFWKGQIAGSGYGVAQFLLYASYALGLWYAAWLVKHGVSDFSRTIRVFMVLMV
SANGAAETLTLAPDFVKGGRAMRSVFETIDRKTEVEPDDVDAAPVPERPKGEVELKHVDFSYPSRPDIQVFRDLSLRARAGKTLA
LVGPSGCGKSSVLALVQRFYEPTSGRVLLDGKDVRKYNLRALRRVVAVVPQEPFLFAASIHDNIAYGREGATEAEVVEAATQANA
HRFISALPEGYGTQVGERGVQLSGGQRQRIAIARALVKQAAIMLLDEATSALDAESERWLFEANLRGPLRRCFWKGQIAGSGYGV
AQFLLYASYALGLWYAAWLVKHGVSDFSRTIRVFMVLMVSANGAAETLTLAPDFVKGGRAMRSVFETIDRKTEVEPDDVDAAPVP
ERPKGEVELKHVDFSYPSRPDIQVFRDLSLRARAGKTLALVGPSGCGKSSVLALVQRFYEPTSGRVLLDGKDVRKYNLRALRRVV
AVVPQEPFLFAASIHDNIAYGREGATEAEVVEAATQANAHRFISALPEGYGTQVGERGVQLSGGQRQRIAIARALVKQAAIMLLD
EATSALDAESERCVQEALERAGNGRTTIVVAHRLATVRNAHTIAVIDDGKVVEQGSHSHLLKHHPDGCYARMLQLQRLTGGAAPG
PPPSSSNGAAA-

Allele: 137 bp deletion in exon 3
Lines: Segaolane, SC757/Marupantse, SC673, and SC648.
MLVGTLGALVHGCSLPVFLRFFADLVDSFGSHANDPDTMVRLVVKYAFYFLVVGAAIWASSWAEISCWMWTGERQSTRMRIRYLD
AALRQDVSFFDTDVRASDVIYAINADAVVVQDAISEKLGNLIHYMATFVAGFVVGFTAAWQLALVTLAVVPLIAVIGGLSAAALA
KLSSRSQDALSGASGIAEQALAQIRIVQAFVGEEREMRAYSAALAVAQKIGYRSGFAKGLGLGGTYFTVFCCYGLLLWYGGHLVR

GNHTNGGLAIATMFSVMIGGLALGQSAPSMAAFAKARVAAAKIFRIIDHRPGISSQDHRAGGQLRLREEHGGVAPREVLRPQRRA
NLAGRA-

Allele: 6 kb deletion spanning exons 4 and 5
Lines: Shan Qui Red and San Chi San
MLVGTLGALVHGCSLPVFLRFFADLVDSFGSHANDPDTMVRLVVKYAFYFLVVGAAIWASSWAEISCWMWTGERQSTRMRIRYLD
AALRQDVSFFDTDVRASDVIYAINADAVVVQDAISEKLGNLIHYMATFVAGFVVGFTAAWQLALVTLAVVPLIAVIGGLSAAALA
KLSSRSQDALSGASGIAEQALAQIRIVQAFVGEEREMRAYSAALAVAQKIGYRSGFAKGLGLGGTYFTVFCCYGLLLWYGGHLVR
GNHTNGGLAIATMFSVMIGGLALGQSAPSMAAFAKARVAAAKIFRIIDHRPGISSRDGEDGGGVELESVTGRVEMRGVDFAYPSR
PDVPILRGFSLSVPAGKTIALVGSSGSGKSTVVSLLERFYDPSA

Allele: 19 bp deletion in exon 5 (predicted peptide of 1240 aa)
Lines: SC937
MLVGTLGALVHGCSLPVFLRFFADLVDSFGSHANDPDTMVRLVVKYAFYFLVVGAAIWASSWAEISCWMWTGERQSTRMRIRYLD
AALRQDVSFFDTDVRASDVIYAINADAVVVQDAISEKLGNLIHYMATFVAGFVVGFTAAWQLALVTLAVVPLIAVIGGLSAAALA
KLSSRSQDALSGASGIAEQALAQIRIVQAFVGEEREMRAYSAALAVAQKIGYRSGFAKGLGLGGTYFTVFCCYGLLLWYGGHLVR
GNHTNGGLAIATMFSVMIGGLALGQSAPSMAAFAKARVAAAKIFRIIDHRPGISSRDGEDGGGVELESVTGRVEMRGVDFAYPSR
PDVPILRGFSLSVPAGKTIALVGSSGSGKSTVVSLLERFYDPSAGQILLDGHDLKSLKLRWLRQQIGLVSQEPTLFATSIKENLL
LGRDSQSATQAEMEEAARVANAHSFIVKLPDGYDTQVGERGLQLSGGQKQRIAIARAMLKNPAILLLDEATSALDSESEKLVQEA
LDRFMIGRTTLVIAHRLSTIRKADVVAVLQGGAVSEMGTHDELMAKGENGTYAKLIRMQEQAHEAALVNARRSSARPSSARNSVS
SPIMTRNSSYGRSPYSRRLSDFSTSDFTLSIHDPHHHHRTMADKQLAFRAGASSFLRLARMNSPEWAYALVGSLGSMVCGSFSAI
FAYILSAVLSVYYAPDPRYMKREIAKYCYLLIGMSSAALLFNTVQHVFWDTVGENLTKRVREKMFAAVLRNEIAWFDADENASAR
VAARLALDAQNVRSAIGDRISVIVQNSALMLVACTAGFVLQWRLALVLLAVFPLVVGATVLQKMFMKGFSGDLEAAHARATQIAG
EAVANLRTVAAFNAERKITGLFEANLRGPLRRCFWKGQIAGSGYGVAQFLLYASYALGLWYAAWLVKHGVSDFSRTIRVFMVLMV
SANGAAETLTLAPDFVKGGRAMRSVFETIDRKTEVEPDDVDAAPVPERPKGEVELKHVDFSYPSRPDIQVFRDLSLRARAGKTLA
LVGPSGCGKSSVLALVQRFYEPTSGRVLLDGKDVRKYNLRALRRVVAVVPQEPFLFAASIHDNIAYGREGATEAEVVEAATQANA
HRFISALPEGYGTQVGERGVQLSGGQRQVKQAAIMLLDEATSALDAESERCVQEALERAGNGRTTIVVAHRLATVRNAHTIAVID
DGKVVEQGSHSHLLKHHPDGCYARMLQLQRLTGGAAPGPPPSSSNGAAA-

Allele: G→A in exon 3 (predicted peptide of 475 aa)
Lines: SC155/R3-80, IS11270, IS11271, SC6/OrangeNo.l_Baijo, and IS12646
MLVGTLGALVHGCSLPVFLRFFADLVDSFGSHANDPDTMVRLVVKYAFYFLVVGAAIWASSWAEISCWMWTGERQSTRMRIRYLD
AALRQDVSFFDTDVRASDVIYAINADAVVVQDAISEKLGNLIHYMATFVAGFVVGFTAAWQLALVTLAVVPLIAVIGGLSAAALA
KLSSRSQDALSGASGIAEQALAQIRIVQAFVGEEREMRAYSAALAVAQKIGYRSGFAKGLGLGGTYFTVFCCYGLLLWYGGHLVR
GNHTNGGLAIATMFSVMIGGLALGQSAPSMAAFAKARVAAAKIFRIIDHRPGISSRDGEDGGGVELESVTGRVEMRGVDFAYPSR
PDVPILRGFSLSVPAGKTIALVGSSGSGKSTVVSLLERFYDPSAGQILLDGHDLKSLKLRWLRQQIGLVSQEPTLFATSIKENLL
LGRDSQSATQAEMEEAARVANAHSFIVKLPDGYDTQVGERGLQLSGGQK-

Allele: 1 bp deletion in exon 3 (predicted peptide of 324 aa)
Lines: SC199/Karad 40581, SC473/Jonar Tamargundi, SC480, SC498, and SC500
MLVGTLGALVHGCSLPVFLRFFADLVDSFGSHANDPDTMVRLVVKYAFYFLVVGAAIWASSWAEISCWMWTGERQSTRMRIRYLD
AALRQDVSFFDTDVRASDVIYAINADAVVVQDAISEKLGNLIHYMATFVAGFVVGFTAAWQLALVTLAVVPLIAVIGGLSAAALA
KLSSRSQDALSGASGIAEQALAQIRIVQAFVGEEREMRAYSAALAVAQKIGYRSGFAKGLGLGGTYFTVFCCYGLLLWYGGHLVR
GNHTNGGLAIATMFSVMIGGLALGQSAPSMAAFAKARVAAAKIFRIIDHRPASPRGTARTAAAWSWSR-

Allele: 22 bp deletion at the intron-exon junction of exon 4 and intron 4 (predicted peptide of 461 aa)
Lines: SC1424

MLVGTLGALVHGCSLPVFLRFFADLVDSFGSHANDPDTMVRLVVKYAFYFLVVGAAIWASSWAEISCWMWTGERQSTRMRIRYLD
AALRQDVSFFDTDVRASDVIYAINADAVVVQDAISEKLGNLIHYMATFVAGFVVGFTAAWQLALVTLAVVPLIAVIGGLSAAALA
KLSSRSQDALSGASGIAEQALAQIRIVQAFVGEEREMRAYSAALAVAQKIGYRSGFAKGLGLGGTYFTVFCCYGLLLWYGGHLVR
GNHTNGGLAIATMFSVMIGGLALGQSAPSMAAFAKARVAAAKIFRIIDHRPGISSRDGEDGGGVELESVTGRVEMRGVDFAYPSR
PDVPILRGFSLSVPAGKTIALVGSSGSGKSTVVSLLERFYDPSAGQILLDGHDLKSLKLRWLRQQIGLVSQEPTLFATSIKENLL
LGRDSQSATQAEMEEAARVANAHSFIVKLPDGYDT-

Allele: T→A in exon 5
Lines: IS19026
MLVGTLGALVHGCSLPVFLRFFADLVDSFGSHANDPDTMVRLVVKYAFYFLVVGAAIWASSWAEISCWMWTGERQSTRMRIRYLD
AALRQDVSFFDTDVRASDVIYAINADAVVVQDAISEKLGNLIHYMATFVAGFVVGFTAAWQLALVTLAVVPLIAVIGGLSAAALA
KLSSRSQDALSGASGIAEQALAQIRIVQAFVGEEREMRAYSAALAVAQKIGYRSGFAKGLGLGGTYFTVFCCYGLLLWYGGHLVR
GNHTNGGLAIATMFSVMIGGLALGQSAPSMAAFAKARVAAAKIFRIIDHRPGISSRDGEDGGGVELESVTGRVEMRGVDFAYPSR
PDVPILRGFSLSVPAGKTIALVGSSGSGKSTVVSLLERFYDPSAGQILLDGHDLKSLKLRWLRQQIGLVSQEPTLFATSIKENLL
LGRDSQSATQAEMEEAARVANAHSFIVKLPDGYDTQVGERGLQLSGGQKQRIAIARAMLKNPAILLLDEATSALDSESEKLVQEA
LDRFMIGRTTLVIAHRLSTIRKADVVAVLQGGAVSEMGTHDELMAKGENGTYAKLIRMQEQAHEAALVNARRSSARPSSARNSVS
SPIMTRNSSYGRSPYSRRLSDFSTSDFTLSIHDPHHHHRTMADKQLAFRAGASSFLRLARMNSPEWAYALVGSLGSMVCGSFSAI
FAYILSAVLSVYYAPDPRYM-

Flanking sequence of causal variants or haplotype-based markers used in KASP marker development

snpSB00493 Sbv3.1_09_57040002W *dw1* (A/T)
CACAGCACCATTGACCCCACCACCTGAACTAGCTCATGCAACAACGCCTTCTTCTCCAGATGTTCCATATGCTCGATTTCTTTCTTCT
TCTATGGATATC[A/T]AAACTGCCAGCAAGGAGCATAACATGCCTTTCTTATCAACAGCCTATTCTGGTGGTTCAGGACTCCAGGCA
TCCTACCCACTTTACCCTGAAAGCCCTTG

snpSB00493 (*dw2*) Sbv3.1_06_42806045I *dw2* (GA/-)
ATTCTGGGCCAATCAAGAAGCTATATGGCTCTGTTGCAGTTCAAATCAACGAGGAGCAAGATGACAAGAGTAAGGCGGGGAAAG
TTTCTATGCTGCCTGA[GA/-]AATTGGCAGGAAGTTCAGTGGGTAAGCCAAGTGGAATCGGTAAGGGGCAGAGCAAGAGCTCGG
CCAAGAAGAATTTGAGATCAGCATCTCCTACCACTGG

snpSB00646 Sbv3.1_07_59821995R *dw3-ref* (haplotype-based marker)
TCTAATGATACTTAGTTGATACCAAAAATATTATTGTTTTACTATATAAATTTGGTCAAACTTAAAAATCTTTGACTCTCCAAGATTCTT
AAAATGACTT[A/G]TAATTTGATACGGAGGGAGTACTCACCGAYAGTAGCMATCATTCATGCRCTACCTTCATTTTTCTGGTCCCTA
TCTGTTGCTGCTATTGCAAATAAGCAG

snpSB00715 Sbv3.1_07_60018674I *dw3-sd6* (haplotype-based marker)
CAAGATTCGATGTGATGGARAATTTTGAAAACTTTTTGGTTTTCAGGAACAAGGCCCGATATCCCCGATCAGCCGACCGGCATCAY
GTGCACGTGTTTCT[C/CA]AACTTTGTGGGAGTACCGTACTCTTTTCCTGTGGACTGTGGTAGCTGGCTTCTGAGGATGGCAAAG
CGACACGTCCACACGATAGTGTATTTTATCATAT

snpSB00716 Sbv3.1_07_59651093Y *dw3-sd6* (haplotype-based marker)
TGGAGTCAGTGCCCGCTCACACTCGTAMGRGACGAGACGGGAGTCGCGATTCGCGAAGATCACTGCTCGCAACTCAAGATGGC
AACGGGTACCCGAAACC[C/T]GAGTACCCGACGGGTTTTACCCGMTAAAGAGAYGGGTATGGAATGAATTTTCTACYCGTGGGT
ATGTTATTGGRCAAGATCCTATACCCATCGGGTATGC

Flanking sequence of new *dw3* alleles identified for future KASP marker development

Allele: G→A (C→T) in exon 3 (predicted peptide of 475 aa)

Lines: SC155/R3-80, IS11270, IS11271, SC6/OrangeNo.l_Baijo, and IS12646

TGAAAAACTTGGCAGTCAAAGTCAACCCGTTTGTTTGACACTGCGTGCATGGCCGGTGCAGGTTGGGGAGCGCGGCCTGCAGC
TCTCCGGCGGGCAGAAG[C/T]AGCGCATCGCCATCGCCCGCGCCATGCTCAAGAACCCTGCCATCCTGCTGCTGGACGAGGCTA
CCAGCGCGCTCGACTCCGAGTCGGAGAAGCTCGTGCA

Allele: 1 bp deletion in exon 3 (predicted peptide of 324 aa)

Lines: SC199/Karad 40581, SC473/Jonar Tamargundi, SC480, SC498, and SC500

CGTCCAGGGCCCTCGGGCAGTCGGCGCCGAGCATGGCCGCGTTCGCCAAGGCGCGCGTGGCGGCCGCCAAGATCTTCCGCATC
ATCGACCACAGGCCGG[G/-]CATCTCCTCGCGGGACGGCGAGGACGGCGGCGGCGTGGAGCTGGAGTCGGTGACGGGGCGGG
TGGAGATGAGGGGCGTGGACTTCGCGTACCCGTCGCGG

Allele: T→A (A→T) in exon 5

Lines: IS19026

TCCCTGGGCTCCATGGTCTGCGGCTCCTTCAGCGCCATCTTCGCCTACATCCTCAGCGCCGTGCTCAGCGTCTACTACGCGCCGGA
CCCTCGCTACATG[A/T]AGCGCGAGATCGCCAAGTACTGCTACCTGCTCATCGGCATGTCCTCCGCGGCGCTGCTGTTCAACACGG
TGCAGCACGTGTTCTGGGACACGGTCGGCGA
