# Supplementary material for: Mining sorghum pangenome enabled identification of new dw3 alleles for breeding stable-dwarfing hybrids
Source: G3 (Bethesda). 2025 Mar 12;15(5):jkaf054. doi: 10.1093/g3journal/jkaf054 (PMC12060247; doi:10.1093/g3journal/jkaf054)
Supplement: jkaf054_Supplementary_Data [file jkaf054_supplementary_data.zip › Supplemental_Figure_Legends_G3-2024-405623.docx]

**Figure S1. Composite interval mapping of sorghum NAM plant height from MN2015.**

Plant height from the MN2015 field trial was used to conduct linkage mapping of individual NAM families. The major sorghum dwarfing loci *dw1*, *dw2*, or *dw3* mapped with the individual NAM families.

**Figure S2. KASP marker genotyping of *dw1*, *dw2*, and *dw3-ref* alleles.**

Casual variant markers were designed to identify *dw1* and *dw2* alleles. Haplotype-based markers were used to differentiate the *dw3-ref* allele. Homozygous desired allele was colored in blue, homozygous alternate allele in red, and the heterozygotes were indicated in purple.
