# Supplementary figures and images for: Mining sorghum pangenome enabled identification of new dw3 alleles for breeding stable-dwarfing hybrids
Source: G3 (Bethesda). 2025 Mar 12;15(5):jkaf054. doi: 10.1093/g3journal/jkaf054 (PMC12060247; doi:10.1093/g3journal/jkaf054)

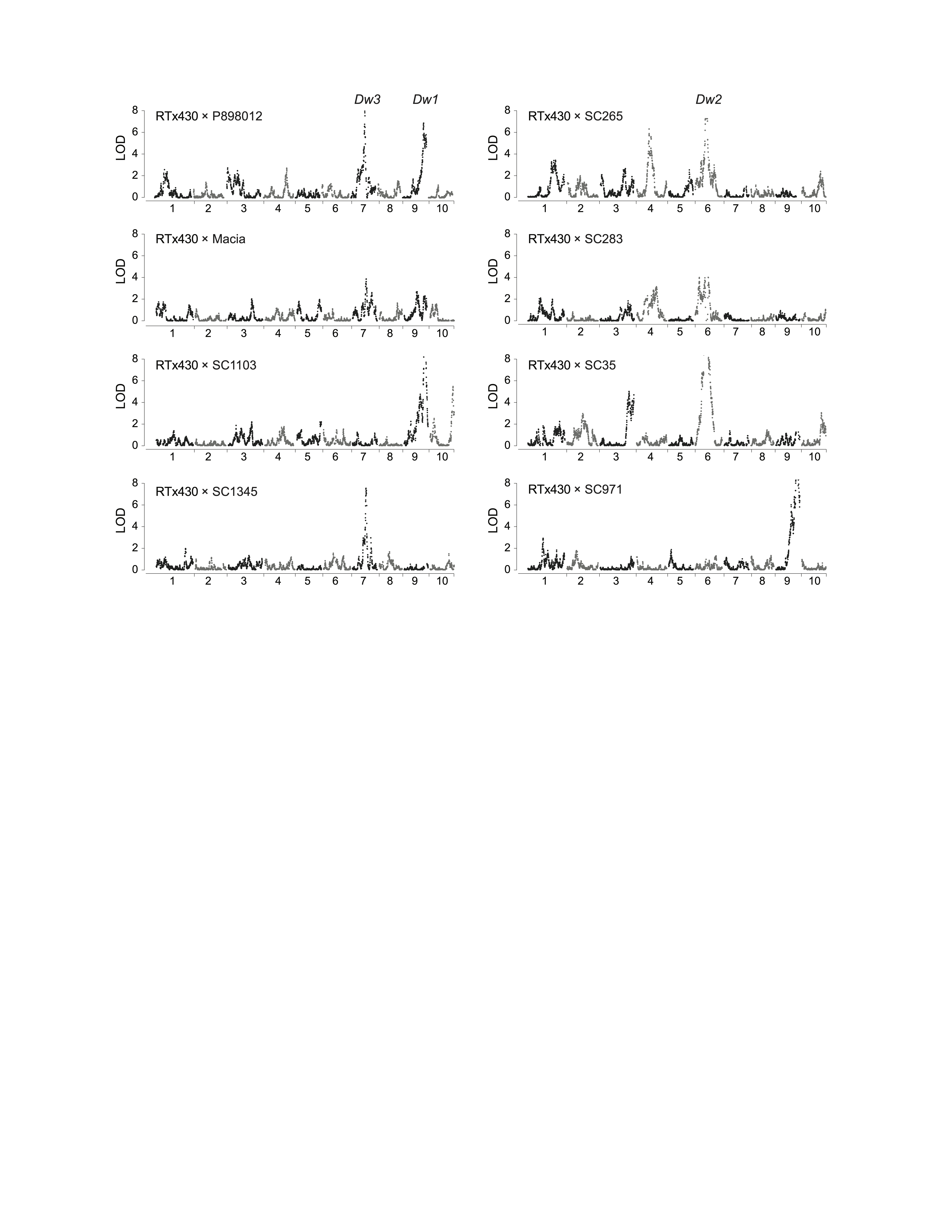

Supplement: jkaf054_Supplementary_Data [file jkaf054_supplementary_data.zip › Figure_S1_G3-2024-405623.tif]

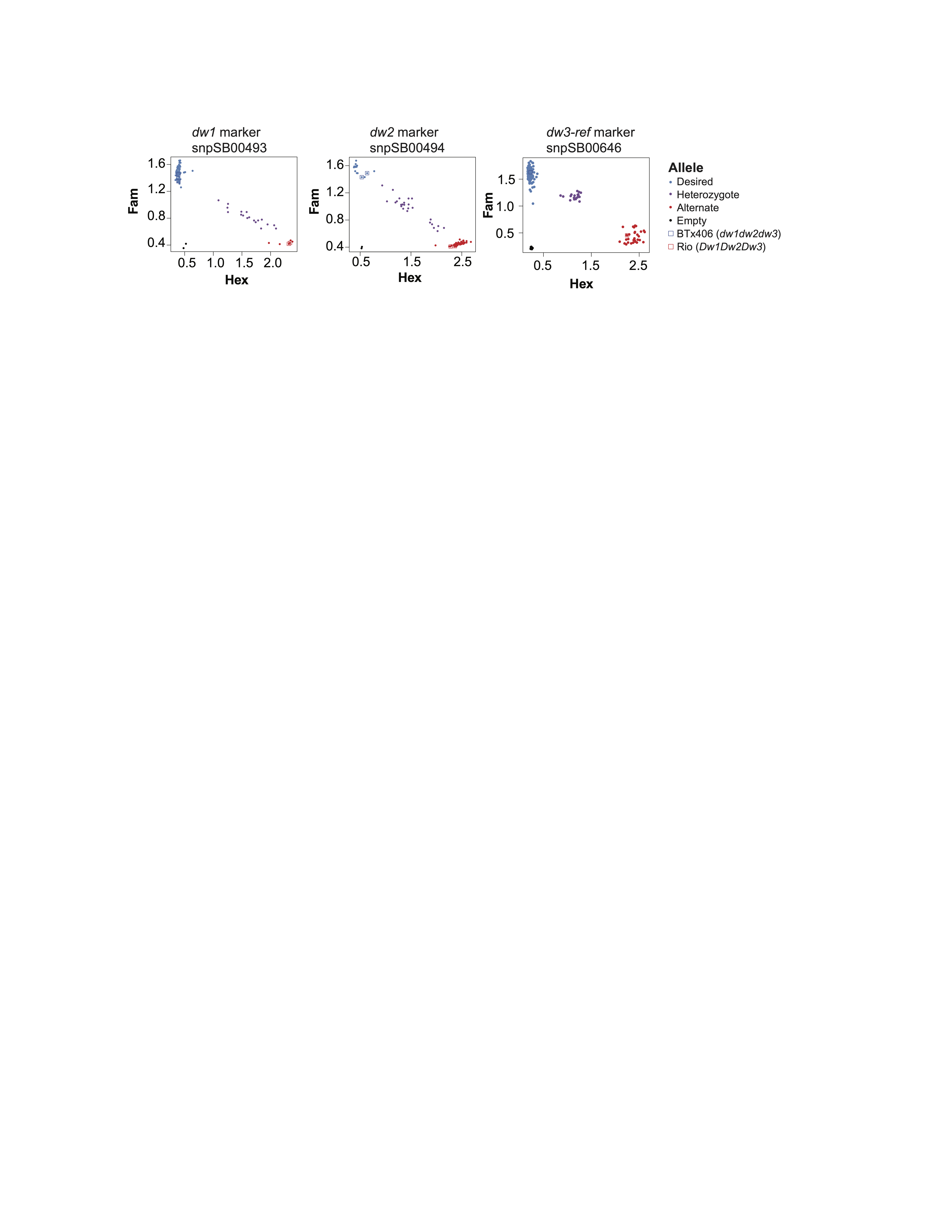

Supplement: jkaf054_Supplementary_Data [file jkaf054_supplementary_data.zip › Figure_S2_G3-2024-405623.tif]
